# Micro X-Ray Computed Tomography Mass Loss Assessment of Different UHMWPE: A Hip Joint Simulator Study on Standard *vs*. Cross-Linked Polyethylene

**DOI:** 10.1371/journal.pone.0170263

**Published:** 2017-01-20

**Authors:** Saverio Affatato, Filippo Zanini, Simone Carmignato

**Affiliations:** 1 Medical Technology Laboratory, Rizzoli Orthopaedic Institute, Bologna—Italy; 2 Department of Management and Engineering, University of Padova, Vicenza—Italy; Universidad de Zaragoza, SPAIN

## Abstract

More than 60.000 hip arthroplasty are performed every year in Italy. Although Ultra-High-Molecular-Weight-Polyethylene remains the most used material as acetabular cup, wear of this material induces over time *in vivo* a foreign-body response and consequently osteolysis, pain, and the need of implant revision. Furthermore, oxidative wear of the polyethylene provoke several and severe failures. To solve these problems, highly cross-linked polyethylene and Vitamin-E-stabilized polyethylene were introduced in the last years. In *in vitro* experiments, various efforts have been made to compare the wear behavior of standard PE and vitamin-E infused liners. In this study we compared the *in vitro* wear behavior of two different configurations of cross-linked polyethylene (with and without the add of Vitamin E) ***vs***. the standard polyethylene acetabular cups. The aim of the present study was to validate a micro X-ray computed tomography technique to assess the wear of different commercially available, polyethylene’s acetabular cups after wear simulation; in particular, the gravimetric method was used to provide reference wear values. The agreement between the two methods is documented in this paper.

## Introduction

Total hip arthroplasty (THA) is a successful orthopaedic standardized procedure to restore functionality on damaged hip due injury or arthritis [1]. As the number of successful operations has increased, the goal of developing alternate bearing surfaces has been to create a joint with decreased friction and wear rates but with increased strength. Moreover, techniques have become standardized and the average age of those receiving hip replacements has reduced. Since ideal bearing surfaces for THA are still being continuously sought, continue effort are made in order to obtain the “ideal bearing surface; the bearing surface should have superior wear characteristics and should be durable, bio-inert, cost-effective, and easy to implant [2].

Future bearing surfaces can be developed by changing the design and improving the polyethylene used as acetabular cup for THA. In *in vitro* research studies, strong efforts have been made to improve the design, the material properties, and the method to improve the wear measurements [3–6]. The most common used measurement method for *in vitro* wear assessment is the gravimetric method, the so-called “gold-standard” measurement practice [7]. The gravimetric method is the standard measurement in which a microbalance is used to measure the mass loss of the specimens during a wear test. The weight loss is then calculated as the difference of the two measurements. On one side this method has proven to be sufficiently accurate to globally quantify the worn material, on the other side it cannot provide information about the distribution of wear over the worn surface and about possible plastic deformations. Recently, coordinate measuring systems (CMSs) such as coordinate measuring machines (CMMs) and micro X-ray computed tomography (micro-CT), have been used as alternatives to overcome these limitations of the gravimetric method [7–12], since they allow evaluating global wear volume, local wear distribution and deformations. In particular, CT has the potential of obtaining a quantitative three-dimensional (3D) information of the entire object geometry in a non-destructive and non-contact way [13, 14]. Moreover, CT can be considered particularly suited for analyzing polymeric components, especially if compared with traditional tactile CMMs, which could induce damages and unwanted deformations to the objects due to clamping and probing forces [15].

In this context, the main goal of this study was to compare gravimetric and micro-CT wear loss measurements of different polyethylene’s acetabular cups [vitamin E-stabilized cross-linked PE, cross-linked PE, and standard Ultra-High-Molecular-Weight-Polyethylene (UHMWPE)].

More in depth, the research objective was twofold:

- designing a new procedure of wear evaluation by micro-CT;
- comparing micro-CT results with the so-called gold standard gravimetric ones.

## Materials and Methods

### Materials

Three different batches of commercial polyethylene acetabular cups (32-mm inner x 50-mm outer diameters; 4 specimens for each batch– 3 in run onto the simulator and one as control-check) coupled with 32-mm metallic femoral heads (cobalt-chromium-molybdenum -CoCrMo). For this investigation we have selected three different types of UHMWPE most used in the orthopaedic field nowadays [16]: vitamin E-stabilized (0,1%) cross-linked PE (hereinafter called XLPE_VE), cross-linked PE (hereinafter called XLPE), and standard bars made of GUR 1020 (Polymax, Adler, Milan, Italy). XLPE acetabular cups were obtained from a cylindrical bar, firstly γ-ray irradiated to 75 KGy (±10%), then thermally processed at 150°C, in order to remove free radicals created during irradiation. Finally, the cups were machined to their final shape. Similarly, XLPE_VE acetabular cups were machined from a Vitamin E-blended UHMWPE bar (Polymax, Adler, Milan, Italy), after electron beam irradiation to 75 KGy (±10%), followed by a thermal treatment at 150°C under nitrogen for 12 hours. All polyethylene acetabular cups were pre-soaked for four weeks prior the wear tests.

### Wear test details

Wear test was performed using a 12-station hip joint simulator (IORSynthe, Bologna, Italy). The simulator utilized hydraulic actuators to apply the cyclic vertical compressive loads (oscillating between 102 and 3000 N applied perpendicular to the acetabular components as recommend by the International ISO 14242). Further details are available on international literature [17]. The lubricant used was 25% (v/v) new born calf serum balanced with distilled water and containing 0.2% sodium azide to retard bacterial growth and 20 mM EDTA (ethylene-diamine-tetracetic acid) to minimize precipitation of calcium phosphate. The length of the wear test was planned at two million cycles (Mc).

### Weight loss by gravimetric method

All the procedures to clean and weight the components were performed following international guidelines (ISO 14242–2) and consolidated internal protocols [17, 18]. The weight loss of the cups (the average between three weights) was determined every 0.4 million cycles using a microbalance (Sartorius Cubis MSE 225 s-000-du, Goettingën, Germany) with a precision of 0.01 mg. Wear trend was determined from the weight loss of each acetabular cup, corrected by acetabular soak control; the wear rates, calculated from the steady-state slopes of the weight loss versus number of cycles lines, were obtained using least squares linear regression. A nonparametric statistical test (Kruskall-Wallis—K-W) was used in evaluating the difference between the three types of liners. In particular, the statistical significance between the different PE was assessed at each weight stop. Statistical significance was set at p < 0.05.

### Procedure for micro-CT wear assessment

Micro-CT geometrical measurements of the acetabular cups were conducted before and after the wear test, so that it was possible to evaluate the volumetric wear. For these measurements, a metrological micro-CT system was used, featuring a 225 kV micro-focus X-ray tube, 2000x2000 pixels flat-panel detector (16bit), temperature-controlled cabinet and maximum permissible error (MPE) for length measurements equal to (9 + L/50) μm (L is the measured length in mm). All CT scans were performed at a controlled temperature of 20 ± 0.2°C. The metrological behavior of the CT system was periodically verified using calibrated objects [19], in order to check and correct measurement errors, including scale errors. In fact, it is well known [19, 20] that temperature control and metrological performance verification, as well as systematic errors correction, are necessary to reduce measurement errors. Before the CT scan, all the acetabular cups (immerged in an appropriate retention liquid), were carefully dried and then kept inside the cabinet of the micro-CT system for 100 minutes, so they could stabilize in terms of temperature (20 ± 0.2°C) and residual liquid content. The source voltage and current were set at 194 kV and 46 μA respectively, with integration time equal to 1415 ms. The duration of each scan was of 35 minutes to obtain 1500 projections of the component while rotating around the axis of the CT system’s rotary table. No physical filtration was applied. The X-ray 2D projections were then used to reconstruct a 3D volume of the investigated components by means of filtered back-projection algorithm (19). The size of the volumetric pixel (voxel) of the reconstructed 3D volume was equal to 31 μm. Adaptive local methods were applied in order to accurately determining the surface of the reconstructed model (19). The volumetric wear of the acetabular cups was achieved by subtraction of volumes computed before and after the wear test. The weight losses to be compared with gravimetric results were calculated by using a density value of 0.934 mg/mm^3^. Finally, after aligning unworn and worn CT volumes, wear maps were generated by the commercial software VGStudio MAX (Volume Graphics GmbH, Germany), which was used to calculate the difference maps between unworn and worn surfaces, showing the local deviations (measured in millimeters) caused by wear tests.

### Comparison between micro-CT and gravimetric method

For a combined graphical/statistical interpretation of the comparison of micro-CT and gravimetric methods, two commonly used approaches were chosen: a scatter plot with correlation and regression analysis and a difference plot (Bland-Altman plot [21]) with 95% limits of agreement. Linear regression quantifies goodness of fit with the coefficient of determination (R^2^). Although the correlation quantifies if two variables are related, a high correlation does not necessary mean a good agreement between the two methods. Thus, the Bland-Altman (B&A) plot was used to describe the agreement between the two quantitative measurements. It consists of a diagram in which the difference of the two measurements is plotted against the mean of the two measurements. To check the assumption of normality of distribution of differences, a Shapiro-Wilk test [22] was done (normality occurs when p > 0.05).

## Results

### Gravimetric wear results

**Fig 1:**
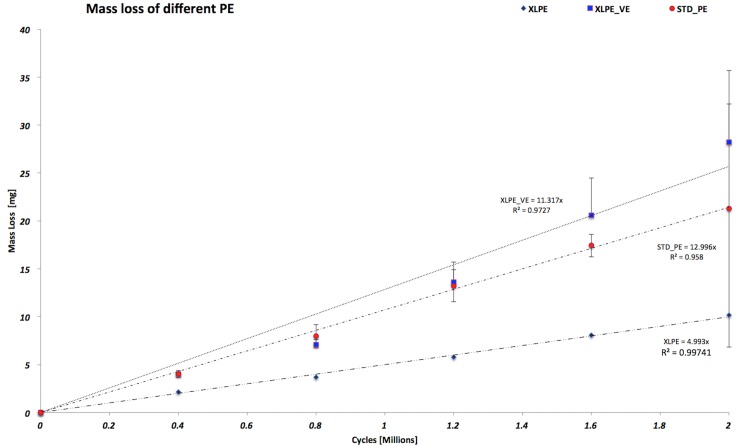
Wear trend of all different configurations of polyethylene.

**Fig 1** shows the wear trend of the different PE configurations. In particular, the XLPE acetabular cups configuration maintained the lowest wear behavior through the wear test.

From this picture is interesting to emphasize that the STD_PE and the XLPE_VE maintained the same wear behavior from 0 to 1.2 million cycles. From 1.2 Mc to the end of the study the XLPE_VE configuration wore more than the STD_PE. On the contrary, the XLPE configuration has shown a wear trend 2 times less than the other two configurations. No statistically significant difference (p = 0.252) was observed between all the three configurations tested at two millions of cycles (**Table 1**).

**Table 1:** Weight loss ± Standard deviation of all specimens tested at two millions of cycles.

	Mean ± Standard deviation			
Cycles [Mc]	STD_PE	XLPE	XLPE_VE	K-W test (*p-value*)
**0.4**	4.0 ±0.4	2.1 ±0.6	4.0 ±0.3	0.066
**0.8**	8.0 ±1.2	3.7 ±1.3	7.1 ±0.5	0.051
**1.2**	13.2 ±1.7	5.8 ±1.2	13.6 ±2.1	0.066
**1.6**	17.4 ±1.2	8.1 ±2.1	20.6 ±3.9	0.051
**2**	21.3 ±14.4	10.2 ±2.1	28.2 ±4.0	0.252

### Micro-CT wear results and comparison with gravimetric method

The volumetric wear of nine acetabular cups (3 specimens for each material type) measured by micro-CT are listed in **Table 2** after conversion to milligrams (mg)–using constant value for UHMWPE density (0.945 g/cm^3^).

**Table 2:** Comparison of gravimetric and CT wear measurements for specimens tested at 2 Mc.

	Weight loss (mg)		
Acetabular cup	CT	Gravimetric	Difference
***XLPE_1***	2.97	5.40	-2.42
***XLPE_2***	4.91	6.86	-1.95
***XLPE_3***	9.83	9.51	0.32
***STD_PE_1***	23.26	20.37	2.89
***STD_PE_2***	21.38	23.06	-1.69
***STD_PE_3***	19.24	17.77	1.47
***XLPE_VE_1***	21.89	23.94	-2.06
***XLPE_VE_2***	13.79	16.43	-2.64
***XLPE_VE_3***	15.04	18.41	-3.37

**Fig 2:**
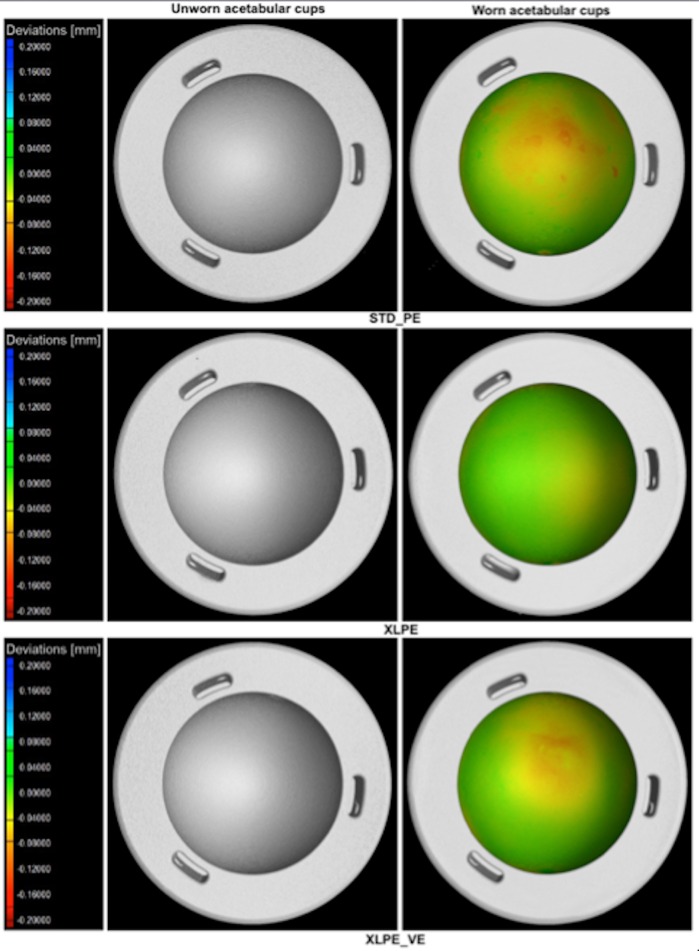
CT reconstructed 3D models of three examples of acetabular cups (one per each material type) obtained by micro-CT scanning at time T = 0 (on the left of the picture) and corresponding wear maps at two millions of cycles (on the right of the picture). The chromatic scales (on the left) indicate the deviations, in mm, between the CT measurements obtained before and after wear; i.e. they represent local wear.

The XLPE acetabular cups are confirmed to be less affected by wear. Local wear distribution mapping on internal calottes of one acetabular cup per each material type is shown as a matter of example on **Fig 2**. This figure is purely meant to show the benefit of acquiring a wear map, which is one of the main advantages of the micro-CT method.

The wear maps show the distribution of wear on each acetabular cup, confirming that the STD UHMWPE has as an higher amount of wear, localized to a specific area that is strictly dependent on the load configuration and positioning adopted during the wear test.

**Fig 3:**
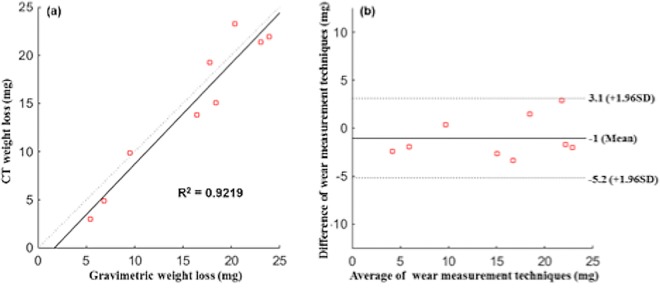
(a) Linear regression analysis between volumetric CT and gravimetric wear evaluations; (b) Bland-Altman plot to evaluate the consistency between CT and gravimetric wear.

**Fig 3(A)** compares CT results with gravimetric ones, showing a linear correlation with a coefficient of determination, R^2^, equal to 0.9219. The Bland-Altman plot illustrated in **Fig 3(B)** demonstrates the agreement between the two measuring techniques. Differences were calculated as CT equivalent weight loss minus gravimetric weight loss. The mean difference (bias) was found to be -1 mg with ±1.96 S.D. (3.1–5.2 mg), which is related to a confidence interval of 95%. A Shapiro-Wilk test (p = 0.14) confirmed the normality of distribution of differences.

## Discussion

The process of cross-linking has been utilized to improve the wear resistance of polyethylene. Some hip simulator studies have shown that the cross-linking process can reduce the type of wear that occurs in acetabular components by >95% [18, 23, 24]. Pre-clinical wear simulations are necessary to predict and quantify the consequent hip prostheses functionality. The aim of the present study was to validate a micro-CT based procedure to assess the wear of commercially available different configurations of polyethylene acetabular cups. In this light, the gravimetric quantification method of wear was considered the reference value. The two methods were found to be correlated and in agreement with mean difference in the order of 11%. The highest percentage difference (≅45%) was registered for the least worn component (XLPE_1). The differences are not only due to the uncertainty of CT wear measurements, but also to the uncertainty of the density value. Furthermore, although the gravimetric method is currently considered the gold standard, it is also unavoidably affected by uncertainties of measurements.

To the author’s knowledge, few reports are available on this matter that uses micro-CT to quantify wear on hip components. Teeter M.G. and al. [25] validated the micro-CT technique for measuring wear volume in polyethylene acetabular liners. In particular, these authors used the micro-CT to measure surface deviation and therefore calculate the volume of wear plus creep of retrieved acetabular liners obtained a maximum 3D deviation of the articular surface of −2.48 ± 0.02 mm, with maximum backside deviation of 0.46 ± 0.02 mm. These authors, in another work [26] observed no significant difference between micro-CT and gravimetric volume measurements for wear volumes higher than 32 mm^3^. In agreement, Engh and al. [27] used a micro-CT technique to quantify the polyethylene wear on retrieved knee components and they found that the micro-CT technique can determine the volume and location of wear of retrieved tibial liners. Recently, Parrilli and co-workers used a micro-CT technique to approach a technique to measure the volume loss of a ceramic femoral head [28]. The new method for wear assessment presented in this work proved to be in good agreement with the standard gravimetric analysis. On the other hand, CT measurements are more time consuming and have higher costs than the gravimetric method. However, CT offers unique possibilities of analysis thanks to the capability of supplying not only the overall wear loss of the component but also detailed and effective information on wear distribution (maps of local wear). This advantage allows discriminating not only how much material the prosthesis has lost but also where wear and/or plastic deformations are localized. Future developments of this work will be focused on determining the uncertainty of CT wear measurements.

## Conclusions

The results obtained from the approach presented in this work produced important improvements on the wear measurement techniques used for the total hip replacement. From the obtained results, it can be concluded that micro-CT and gravimetric measurements of wear are in agreement, provided that micro-CT dimensional measurements are performed under temperature control and that metrological performance verification and systematic errors correction are adopted to allow metrological use of micro-CT. Despite this adopted methodology for wear loss characterization requires further works in order to improve its reliability and accuracy, it has the potential to become a new reference method in wear assessment of UHMWPE components. In fact, the micro-CT method can be useful to determine the location of wear and to analyze how wear distribution for the investigated specimens change by the effect of different loads.
